# A new dataset of pheromone and pheromone-gene structures from the ciliate, *Euplotes crassus*

**DOI:** 10.1016/j.dib.2023.109430

**Published:** 2023-07-20

**Authors:** Graziano Di Giuseppe, Claudio Alimenti, Pierangelo Luporini, Adriana Vallesi

**Affiliations:** a Department of Biology, University of Pisa, Italy; b MARinePHARMA Center, University of Pisa, Italy; c Laboratory of Eukaryotic Microbiology and Animal Biology, School of Bioscience and Veterinary Medicine, University of Camerino, Italy

**Keywords:** Water-borne protein pheromones, Ciliate multiple mating-type systems, Macronuclear gene-size DNA molecules, Ciliate conjugation, Sex in protozoan ciliates

## Abstract

Like many other organisms, ciliates communicate and interact socially via diffusible chemical signals, named pheromones, that are functionally associated with a genetic mating-type mechanism of cell self/not-self recognition. In *Euplotes* species, pheromones form species-specific families of small, globular, and disulfide-rich proteins folding into exclusively helical secondary structures. Each is specified by one of a series of high-multiple alleles that are inherited in Mendelian fashion with relationships of co-dominance at the so-called *mat* genetic locus of the cell transcriptionally inert micronuclear genome, and expressed in the transcriptionally active macronuclear genome as individual DNA molecules in which the central coding region is flanked by 5’-leader and 3’-trailer noncoding regions ending with C_4_A_4_/T_4_G_4_ telomeric repeats. In *E. crassus*, a cosmopolitan marine species with a long tradition in the study of ciliate mating systems and breeding patterns, oligonucleotides specific to amino acid sequences of pheromones E*c*-1 and E*c*-α were previously used to clone and sequence a first set of four structurally distinct macronuclear (*mac*) pheromone coding genes, *mac-ec-α, mac-ec-1, mac-ec-2* and *mac-ec-3*, from two interbreeding strains, L-2D and POR-73. The use of these oligonucleotides in PCR amplifications of macronuclear DNA preparations from three other *E. crassus* interbreeding strains, ES10, Fava4 and MN4, has now resulted in the characterization of a second set of eight new pheromone coding genes, *mac-ec-β, mac-ec-γ, mac-ec-δ, mac-ec-ε, mac-*ec-*µ, mac-ec-4, mac-ec-5* and *mac-ec-6*. Multiple alignment between previously and newly determined pheromone-gene sequences reinforces the concept that the *E. crassus* pheromone-gene family includes two sub-families, which likely reflect a duplication of the micronuclear *mat* gene locus and represent an apomorphic trait of the *E. crassus* clade. Members of one sub-family (each identified with a Greek letter) show a 500-bp 5’-leader noncoding region rich in AGGA/AGGGA repetitions, and encode 56-amino acid pheromones with eight conserved Cys residues. Members of the other sub-family (each identified with an Arabic numeral) show an 800-bp 5’-leader noncoding region without AGGA/AGGGA repetitions, and encode 45-amino acid pheromones with ten conserved Cys residues.

**Specifications Table**

**Table:** 

Subject	Biological Sciences
Specific subject area	Eukaryotic Microbiology, Protistology/Protozoology, Molecular/Cell Biology
Type of data	Two tables and three figures
How the data were acquired	Data were obtained from Sanger sequencing of polymerase chain reaction (PCR)-purified products.
Data format	Raw Analyzed
Description of data collection	*Euplotes* strains used in this study were taxonomically identified as *E. crassus* on the basis of morphological and genetic criteria [1]. Macronuclear DNA preparations were amplified using primers designed on conserved sequences adjacent to the telomeric ends and previously determined from other *E. crassus* pheromone coding genes [2]. Amplicons were cloned, plasmids of five distinct clones were Sanger-sequenced in both directions, and partial sequences were overlapped to produce the complete gene sequences.
Data source location	Living cell samples and DNA preparations from *E. crassus* strains source of the analyzed pheromone coding genes are available from the G.D.G. laboratory at the Department of Biology of Pisa University, and the A.V. laboratory at the School of Biosciences and Veterinary Medicine of Camerino University.
Data accessibility	Repository name: Mendeley data https://data.mendeley.com/datasets/63m8bp8zxc
Related research article	A. Vallesi, C. Alimenti, S. Federici, G. Di Giuseppe, F. Dini, G. Guella, P. Luporini, Evidence for gene duplication and allelic codominance (not hierarchical dominance) at the mating-type locus of the ciliate, *Euplotes crassus*. J. Eukaryot. Microbiol. 61 (2014), 620-629. https://doi.org/10.1111/jeu.12140

## Value of the Data

• The mating-type mechanism that controls ciliate conjugation is basically a phenomenon of cell self/not-self recognition driven by cell interactions with diffusible protein pheromones. The determination of new pheromone and pheromone-gene sequences from *E. crassus* improves the knowledge of the molecular basis of this mechanism.
• In *E. crassus*, pheromones are secreted in tiny amounts making it hard to determine their complete amino acid sequences in a significant number via direct chemical analysis of native protein preparations. The PCR primers reported in the dataset are designed on conserved stretches of the pheromone-gene sequences, and help to circumvent the chemical way by cloning and sequencing other pheromone coding genes from new *E. crassus* strains.
• An upgraded dataset of pheromone and pheromone-gene sequences may be used for a more reliable identification of which pheromone structural specificities are functionally more relevant in diversifying conspecific cells in their spectrum of mating interactions, and which nucleotide sequence stretches turn out to be useful in designing new primer combinations for qPCR analyses of pheromone-gene amplification and expression.
• The knowledge of more *E. crassus* pheromone amino acid sequences greatly validates the application of the AlphaFold AI system in scouting their three-dimensional folding, and the knowledge of this folding pattern may add a relevant brick to the general picture of how the *Euplotes* pheromone molecular structures—so far determined only from *E. nobilii, E. petzi* and *E. raikovi* [3,4]—evolve in concert with speciation and habitat colonization.

## 1 Objective

Results from traditional Mendelian analyses of the *E. crassus, E. minuta* and *E. vannus* mating systems have for long accounted for an eccentric model of serial dominance relationships between single-locus multiple *mat*-alleles [[5], [6]^_^^7^], and the synthesis of insoluble (membrane-bound) protein pheromones [8]. Results from two original research articles, based on molecular approaches to the *E. crassus* mating system, conflicted with this model [2,9]. They provided evidence that (i) *E. crassus* cells conform with the more common (basic) ‘*E. patella*-model’ in relation to *mat*-allele relationships of co-dominance and the synthesis of diffusible pheromones [10], and (ii) the difference resides in the evolution of two structurally distinct pheromone-coding gene sub-families likely reflecting a phenomenon of *mat*-gene locus duplication [2]. In addition to reinforcing evidence that a *mat*-gene locus duplication is a distinctive (apomorphic) feature of the *E. crassus* clade, the knowledge of new *E. crassus* pheromone and pheromone-gene sequences may help (i) planning *in silico* analyses of whether and how pheromones from the two sub-families interact in binding target cells, and (ii) exploring whether and how these interactions can account for the serial-dominance relationships that were originally presumed to govern *mat*-allele inheritance and expression in the species of the *E. crassus* clade.

## 2 Data Description

Table 1 reports the denominations of all the pheromone-coding genes that have been cloned from *E. crassus* and analyzed for their sequences, while Table 2 enumerates the respective GenBank sequence numbers. Figs. 1 and 2 illustrate the multiple alignments of the nucleotide sequences that are overall known from *E. crassus* for each of the two pheromone-gene subfamilies: one (Fig. 1) including genes (labelled with progressive Arabic numbers) that show closer orthologous relationships with pheromone genes of other *Euplotes* species; the second (Fig. 2) including genes (labelled with Greek letters) with closer *E. crassus* specificity. Fig. 3 illustrates the multiple amino acid sequence alignments of the cytoplasmic pheromone precursor forms (pre-pro-pheromones) that are specified by the structurally determined sequences of genes from the two sub-families.

**Table 1:** Denominations, encoded pheromones and source strains of previously (light letters) and newly (bold letters) characterized *E. crassus* macronuclear pheromone genes.

*Pheromone genes*	*Encoded pheromones*	*Source strains*	*Collection sites*	*Latitude/Longitude*	*Year*
*mac-ec-α, mac-ec-1*	E*c*-α, E*c*-1	L-2D	Leghorn (IT)	N 43°33’/E 10°19’	1978
*mac-ec-α, mac-ec-2, mac-ec-3*	E*c*-α, E*c*-2, E*c*-3	POR-73	Porto Recanati (IT)	N 43°26’/E 13°40’	1978
***mac-ec-β, mac-ec-4***	**E*c*-β, E*c*-4**	MN4	Montecristo Island (IT)	N 42°20’/E 10°19’	1978
***mac-ec-γ, mac-ec-δ, mac-ec-5***	**E*c*-γ, E*c*-δ, E*c*-5**	Fava4	Favone, Corsica Island (FR)	N 41°46’/E 9°23’	2002
***mac-ec-ε, mac-ec-µ, mac-ec-6***	**E*c*-ε, E*c*-µ, E*c*-6**	ES10	Motril (ES)	N 36°44’/W 3°31’	1986

**Table 2:** GenBank accession numbers of *E. crassus* macronuclear pheromone genes. Genes with previously determined sequences are in light letters, and genes with newly determined sequences are in bold letters.

Gene denominations	Accession numbers
*mac-ec-α*	HM072429
*mac-ec-1*	HM072430
*mac-ec-2*	KF905230
*mac-ec-3*	KF905231
***mac-ec-β***	**OQ630543**
***mac-ec-4***	**OQ630548**
***mac-ec-γ***	**OQ630544**
***mac-ec-δ***	**OQ630545**
***mac-ec-5***	**OQ630549**
***mac-ec-ε***	**OQ630546**
***mac-ec-µ***	**OQ630547**
***mac-ec-6***	**OQ630550**

**Fig. 1:**
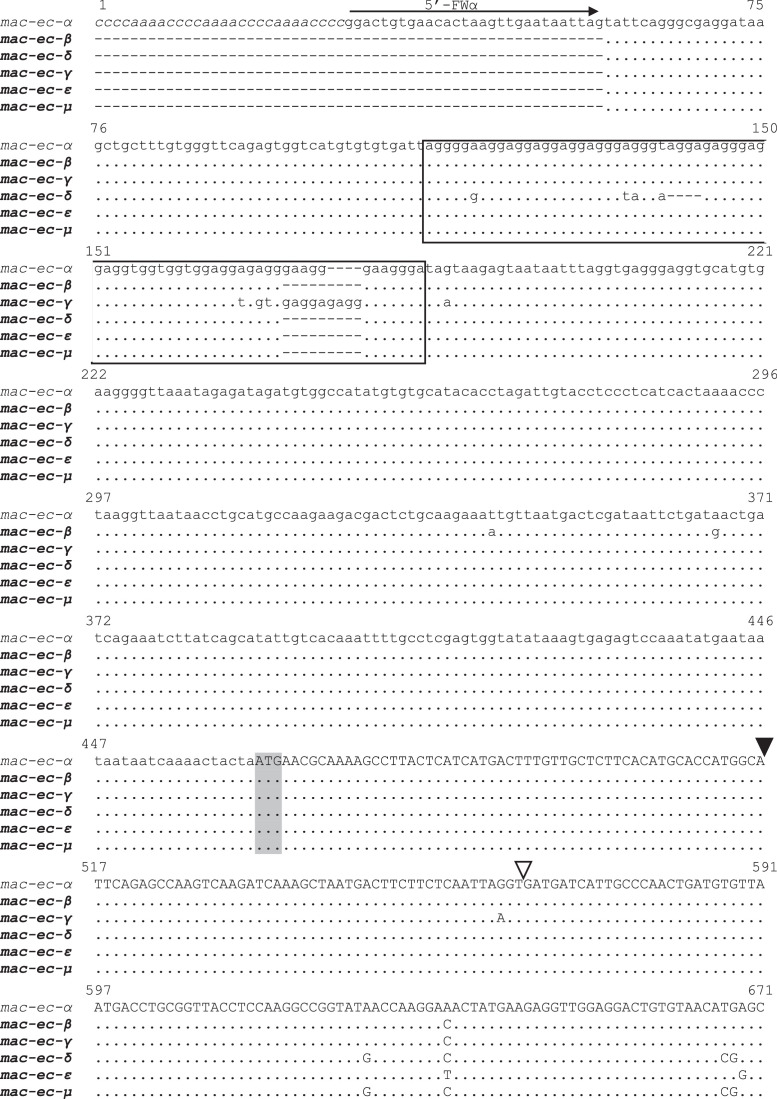

**Fig. 1:**
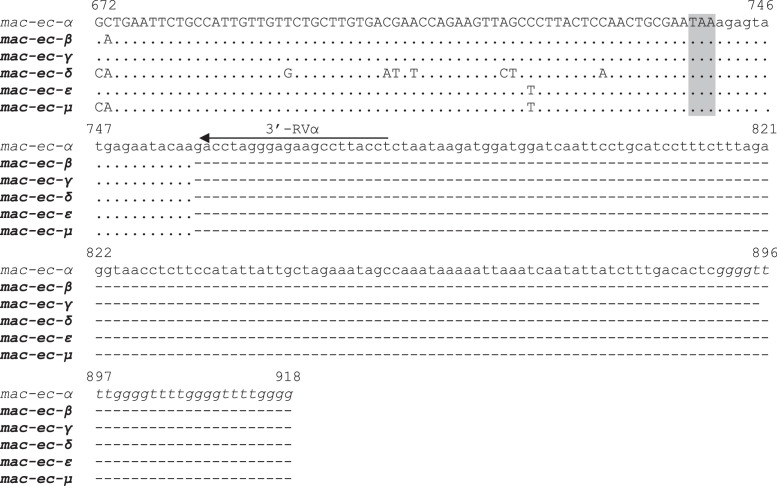
Multiple nucleotide sequence alignment of *E. crassus* pheromone genes of the subfamily (distinguished by Greek letters) encoding pheromone sequences of 56 amido acids with eight Cys residues. Gaps were inserted to optimize the alignment, and dots indicate identical nucleotides. A previously determined sequence is in light letters, and the newly determined sequences are in bold letters. The 5’-leader and 3’-trailer non-coding regions are in lower case letters, the coding region is in capital letters, and the telomeric repetitions are in italics. The ATG start codon and the TAA stop codon are shadowed. Filled and light arrowheads delimit the sequence segments specifying the signal peptide and the pro segment, respectively, of the cytoplasmic (immature) pheromone precursor form (pre-pro-pheromone). In the 5’-leader region, a box delimits the domain characterized by AGGA/AGGGA repetitions.

**Fig. 2:**
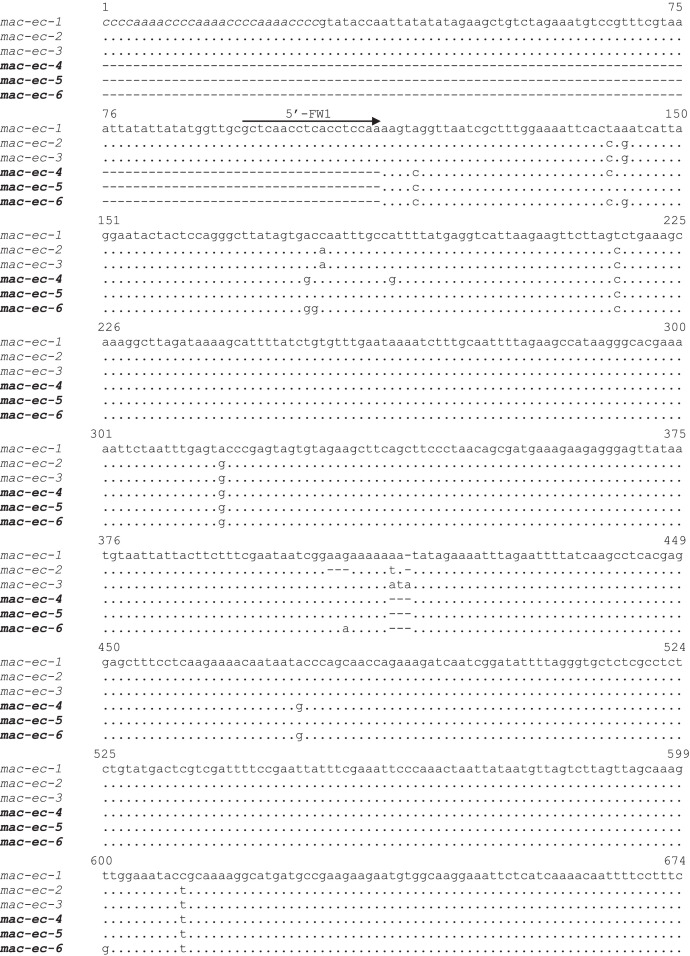

**Fig. 2:**
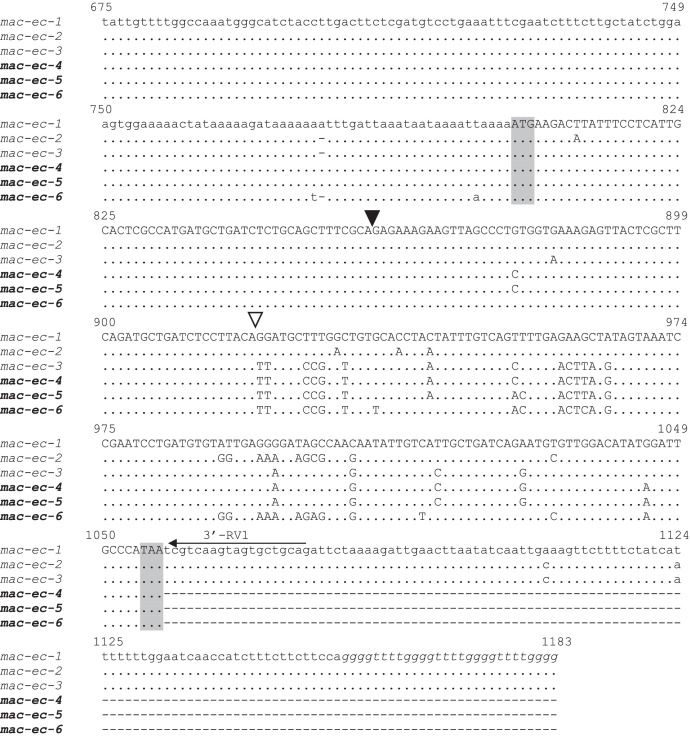
Multiple nucleotide sequence alignment of *E. crassus* pheromone genes of the subfamily (distinguished by Arabic numerals) encoding pheromone sequences of 45 amido acids with ten Cys residues. Gaps were inserted to optimize the alignment, and dots indicate identical nucleotides. Three previously determined sequences are in light letters, and the newly determined sequences are in bold letters. The 5’-leader and 3’-trailer non-coding regions are in lower case letters, the coding region is in capital letters, and the telomeric repetitions are in italics. The ATG start codon and the TAA stop codon are shadowed. Filled and light arrowheads delimit the sequence segments specifying the signal peptide and the pro segment, respectively, of the cytoplasmic (immature) pheromone precursor form (pre-pro-pheromone).

**Fig. 3:**
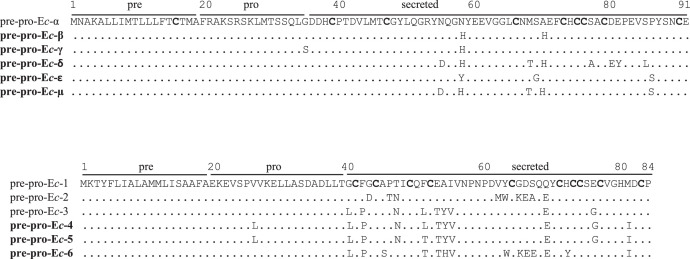
Multiple alignment of pre-pro-pheromone amino acid sequences specified by the two *E. crassus* pheromone-gene sub-families. Bars span the pre, pro and secreted regions, Cys residues are highlighted in bold, and dots indicate identical residues. Previously and newly determined sequences are distinguished by denominations in light and bold letters, respectively.

## 3 Experimental Design, Materials and Methods

### 3.1 Cell Cultures

The *E. crassus* strains used for the pheromone-gene sequence determination were expanded each starting from a single specimen isolated from samples of shallow water and sediment. They were cultivated in sterilized natural, or artificial seawater, at 20-22°C under a light-dark cycle following routine procedures [2] and using the green algae *Dunaliella tertiolecta* as standard food source.

### 3.2 DNA Preparation and PCR Amplification

DNA was prepared, following a standard protocol [1,2], from *E. crassus* cultures previously deprived of food for 2 days and resuspended in fresh seawater for 1 day. Amplifications were run in a GeneAmp 9700 thermal cycler (PE Applied Biosystems) following a standard program (30s 94°C, 40s 58°C, 60s 72°C for 35 cycles), and using oligonucleotides synthesized by Invitrogen (Van Allen Way, Carlsbad, CA). The two oligonucleotides, 5’-FW1 (GCTCAACCTCACCTCCAA) and 3’-RV1 (CTGCAGCACTACTTGACGA), were used as forward and reverse primers, respectively, to amplify the *mac-ec-4, mac-ec-5* and *mac-ec-6* genes. The *mac-ec-β, mac-ec-γ, mac-ec-δ, mac-ec-ε*, and *mac-ec*-*µ* genes were instead amplified with the two oligonucleotides, 5’-FWα (GGACTATGAACACTAAGTTGAATAATTAG) and 3’-RVα (AGGTAAGGCTTCTCCCTAGGTC), used as forward and reverse primers, respectively. The 50-μl reaction mixtures contained 0.5 μg of DNA, 2 mM MgCl_2_, 250 mM of dNTP, one unit of Taq DNA polymerase (Polymed, Florence, Italy), and 0.2 mM of each primer. Amplicons were cloned into the pCR 2.1-TOPO vector of the TOPO TACloning Kit (Invitrogen), and five clones were analyzed for each gene sequence determination.

## Ethics Statements

This work did not involve experimental analyses of animals or humans.

## CRediT authorship contribution statement

**Graziano Di Giuseppe:** Conceptualization, Investigation, Data curation, Writing – original draft. **Claudio Alimenti:** Investigation, Formal analysis. **Pierangelo Luporini:** Conceptualization, Supervision, Writing – review & editing. **Adriana Vallesi:** Conceptualization, Investigation, Data curation, Supervision, Writing – original draft.

## Data Availability

Pheromone and pheromone-gene sequences from the marine ciliate, *Euplotes crassus* (Original data) (Mendeley Data)
